# Extraction of isoflavones from *Puerariae lobata* using subcritical water

**DOI:** 10.1039/c8ra02653j

**Published:** 2018-06-20

**Authors:** Hongguang Zhang, Shuhua Liu, Huaizhi Li, Fumin Xue, Shuxin Han, Liang Wang, Yan Cheng, Xiao Wang

**Affiliations:** Key Laboratory of TCM Quality Control Technology, Shandong Analysis and Test Center, Qilu University of Technology (Shandong Academy of Sciences) Jinan 250014 China wangx@sdas.org chengyan99@163.com; College of Chemistry and Chemical Engineering, Ocean University of China Tsingtao Shandong 266100 China; The People's Hospital of Junan Linyi Shandong 276600 China

## Abstract

As an alternative to organic solvents, subcritical water was employed for the first time as an effective solvent for the extraction of isoflavones from *Puerariae lobata*. Optimum experimental conditions for the extraction of the four main isoflavones were established by single factor experiments, and the optimum experimental conditions for total isoflavone extraction were established further by response surface methodology. With an extraction time of 45 min and a liquid/solid ratio of 1 : 20, the extraction yields of puerarin, 3′-methoxypuerarin, and daidzin reached maxima at extraction temperatures of 120 °C, 140 °C and 200 °C, respectively. Moreover, puerarin, 3′-methoxypuerarin and daidzin were degraded and produced various byproducts due to hydrothermal reactions at higher temperatures. The maximum extraction yields of the total isoflavones were obtained by response surface methodology (extraction time of 45 min, solid/liquid ratio of 1 : 15 and extraction temperature of 120 °C). Compared to conventional solvents, subcritical water utilized less solvent and required a shorter extraction time.

## Abstract 2

Four main isoflavones from *Puerariae lobata* were successfully extracted using subcritical water. Maximum yields of total isoflavones by response surface methodology were obtained.

## Graphical abstract

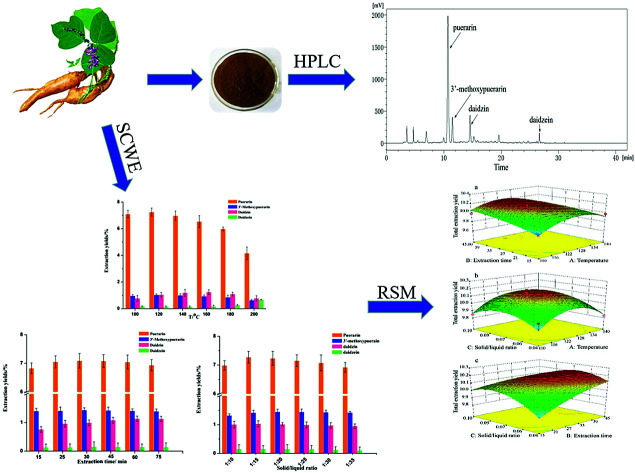

## 1. Introduction

*Puerariae lobata* is widely used as a soup ingredient in the southern part of China. It is also a major pharmaceutical resource for clinical applications. In 2002, *Puerariae lobata* was approved for use in food and drugs by the Ministry of Health in China.^1^ It has been reported as an antioxidant,^2^ anti-metabolic agent^3^ and anticancer agent,^4^ and it can also be used to treat diabetes and cardiovascular diseases.^5^ Reportedly, the major active ingredients in *Puerariae lobata* are isoflavone compounds, such as puerarin, 3′-methoxypuerarin, daidzin and daidzein.^6^ They are widely present in soybean foods^7^ and traditional herbs including *Puerariae lobata*.^8^ The isoflavones are a group of naturally occurring polyphenolic compounds with a variety of physiological activities. In particular, puerarin and 3′-methoxypuerarin have shown anticancer, antioxidant and potential antidiabetic properties.^9^ Additionally, animal studies have suggested that puerarin has a preventive effect on alcohol-induced osteonecrosis.^10^ Besides, the isoflavones are quickly converted to nutrients and easily absorbed into the body.^11–13^ Therefore, the analysis of the four main isoflavone compounds in *Puerariae lobata* is of prime importance.

Methanol and ethanol, which possess strong volatility and toxicity, are traditionally used as organic solvents to efficiently extract isoflavones from *Puerariae lobata* on a large scale.^4,14–16^ These extraction solvents are not environmentally friendly. Moreover, the extraction processes may easily leave solvent residues in the extracts. Water is a safer, cheaper and more easily available solvent than organic solvents. Furthermore, the physicochemical properties of water can be easily manipulated by changing the temperature or pressure.^17^ When water is heated under sufficient pressure to maintain it in a liquid state (*i.e.*, pressurized hot water), there are significant changes to its polarity. Typically, the dielectric constant (*ε*) of water at 25 °C is approximately 80, but it reduces to approximately 27 at 250 °C, which is similar to that of methanol (*ε* = 33 at 298 K) or ethanol (*ε* = 25 at 298 K).^18^ The lower surface tension and viscosity of pressurized hot water also increase mass transfer rates of active compounds from the plant tissue matrix. These physicochemical properties of water are the basis for using subcritical water to replace organic solvents in extraction processes. Thus, subcritical water extraction (SCWE) has received significant attention from researchers over the past three decades as a feasible green alternative to organic solvents.^19,20^ Many extraction studies from herbs or food byproducts using SCWE have been reported. Ibaňez *et al.* investigated the effect of temperatures ranging from 25 to 200 °C on the selective extraction of antioxidant compounds from rosemary leaves.^21^ Subcritical water could extract most of the active compounds from rosemary, such as carnosol, rosmanol, carnosic acid, and methyl carnosate. Some extracts such as cirsimaritin and genkwanin showed high selectivity. Myong-Soo *et al.* studied SCWE of the flavonol quercetin from onion skin using a temperature range of 100–190 °C and extraction times of 5–30 min.^14^ Using SCWE, the quercetin yield was over eight-, six-, and four-fold greater than those obtained using the ethanol, methanol and water-at-boiling-point extraction methods, respectively. Depending on the SCWE conditions, it was possible to obtain targets with different chemical compositions.

Response surface methodology (RSM) is an effective statistical technique for optimizing multiple, inter-related parameters.^22–24^ It is less laborious and time-consuming than other methods that have been applied to process optimization. Therefore, it has been widely used to optimize the extraction processes of active compounds from herbs or food byproducts.^25,26^

This study investigated subcritical water as a feasible processing solvent for extraction of four isoflavone components and total isoflavones from *Puerariae lobata*. SCWE parameters, such as extraction temperature, extraction time and solid/liquid ratio, were systematically optimized by RSM for enhancing the extraction efficiency. The objective of the present work was to achieve rapid, and environmentally friendly water extraction conditions for four isoflavone components and the total isoflavonoids from *Puerariae lobata*.

## 2. Materials and methods

### 2.1 Chemicals and reagents

Reference standards of puerarin, 3′-methoxypuerarin, daidzin and daidzein (purity ≥ 98%) were supplied by Shanghai Yuanye Biotechnology Co., Ltd, China. Methanol and formic acid (Analytical Grade, Tianjin, China) were purchased from Tianjin Kermel Chemical Reagent Co., Ltd. Acetonitrile (Tedia Company Inc., Fairfield, USA) was used for HPLC analysis. Deionized water (>18 MΩ cm) was prepared using an Elga Purelab water system (Elga, England).

Dry *Puerariae lobata* was purchased from the Zhonglu Hospital. The roots of *Puerariae lobata* were washed, dried and ground in a grinder (Zhongxing Weiye, Beijing, China). The powder was passed through a 60 mesh sieve and stored at −4 °C.

### 2.2 Experimental design and statistical analysis

The experiments were executed in two phases. The objective of phase I was to obtain the optimum extraction conditions: extraction temperature, extraction time and ratio of solid/liquid for four isoflavone components in *Puerariae lobata*. The main role of the pressure was to maintain the water in liquid form at high temperature. Therefore, the appropriate pressure was maintained for each given temperature. In our study, water was mainly present in liquid form in our investigated temperature range. In this phase, subcritical water with a temperature range of 100–200 °C was investigated at 20 °C intervals. The extraction temperature of 120 °C was selected for a range of extraction times from 15 min to 75 min. The reason for the selection of 120 °C is described in the Results and discussion. Extraction yields of puerarin, 3′-methoxypuerarin, daidzin and daidzein were determined from the corresponding standard curves obtained by HPLC analysis. Stock solutions of puerarin, 3′-methoxypuerarin, daidzin and daidzein standards were prepared in methanol at concentrations of 60 μg mL^−1^. Working standard solutions were prepared in the range of 3–15 μg mL^−1^ by diluting the appropriate stock solution with methanol, and were stored at −4 °C in darkness. The objective of phase II was to obtain the optimum extraction conditions (extraction temperature, extraction time and ratio of solid/liquid) for the total isoflavones in *Puerariae lobata* by using RSM. On the basis of the results of phase I, the phase II experiment for total isoflavones was conducted over a narrower range of extraction temperatures (110 °C, 120 °C and 140 °C) and extraction times (15, 30 and 45 min) at three different solid/liquid ratios (1 : 10, 1 : 15 and 1 : 25). To further study the interactions between the extraction parameters, RSM was employed to obtain the optimum operating parameters. The Box–Behnken program was used to design experimental projects, analyze statistical data and calculate the regression model. The statistical significance was checked using an *F*-test. The experimental extraction yields of total isoflavones were compared with those predicted from the RSM.

### 2.3 Extraction procedures

#### 2.3.1 Subcritical water extraction

A 10 mL cylindrical stainless steel high-pressure batch extractor with an XTD-7000 isothermal furnace (Haian, China) was used for extraction of isoflavones from *Puerariae lobata*. A 0.20 g sample of dry *Puerariae lobata* powder was extracted with subcritical water in an extractor for the designated time at the designated temperature. Then the extractor was taken out of the furnace, cooled in water and opened. Extracts were collected with methanol. We paid much attention to the selection of the methanol solvent to dissolve the four isoflavones. On the one hand, the dielectric constant of water at 200 °C is similar to that of methanol. When the extracts were cooled to room temperature, we hypothesized that the utilization of methanol with its low dielectric constant might prevent the target extracts from insolubility. On the other hand, some researchers have utilized methanol to extract the isoflavone compounds^27,28^ and we could refer to their experimental procedures. Methanol was added to make the required final volume and the solution was filtered through a 0.45 μm Nylon syringe filter (Jinteng, Tianjin) for HPLC analysis.

#### 2.3.2 Reflux extraction

Zhu^28^ has previously investigated the influence of extraction temperature, time and ratios of ethanol–water, and described a reflux extraction procedure at an extraction temperature of 90 °C, extraction time of 120 min and extraction solvent of 70% ethanol–water. We referred to these published experimental conditions in developing a typical reflux extraction procedure as follows: 1.00 g aliquot of dry *Puerariae lobata* powder was extracted with 30 mL 70% ethanol–water solvent at 90 °C for 120 min in a round-bottom flask with a Dimroth condenser in a thermostatic waterbath. Extracts were transferred into a 100 mL volumetric flask and methanol was added to make the final volume. This extraction procedure was repeated three times.

#### 2.3.3 Ultrasonic extraction

Li^29^ has investigated the influence of temperature and time for ultrasonic extraction and the optimal extraction procedure was confirmed to be 50 °C and 45 min. Thus we used a typical extraction procedure with ultrasonic extraction as follows: 0.20 g dry *Puerariae lobata* powder was extracted with distilled water in an ultrasonic water-bath at 50 °C for 45 min (KQ-600 KDE, Kunshan, China). The extracts were combined and then centrifuged at 10 000 rpm for 10 min. This extraction procedure was repeated three times.

### 2.4 HPLC apparatus and operating conditions

An UltiMate 3000 liquid chromatography system (Thermo Fisher Scientific Inc., Waltham, USA) with a five binary gradient pump, UV detector, column oven and autosampler was used for detection of puerarin, 3′-methoxypuerarin, daidzin and daidzein. Each sample solution (10 μL) was injected into a C 18 column (25 cm × 4.6 mm, 5 μm, InertSustain). Detection was at 260 nm and 35 °C. The mobile phase consisted of two components: (A) 0.1% formic acid in water, and (B) acetonitrile. The gradient elution program was as follows: 15–30% B (0–16 min), 30–100% B (16–34 min), 100–100% B (34–40 min), 100–15% B (40–41 min), 15–15% B (41–42 min). The flow rate was 0.80 mL min^−1^.

### 2.5 Statistical analysis

Calibration curves of puerarin, 3′-methoxypuerarin, daidzin and daidzein were used for calculating the extraction yields. The optimum SCWE conditions, *i.e.*, extraction temperature, extraction time and ratio of solid/liquid from each experiment were chosen based on the highest isoflavone contents. Three replicates of each analysis were performed in order to determine the reproducibility of the procedure. Results are expressed as mean ± SD. Statistical comparisons were made by one-way analysis of variance (ANOVA). Differences were considered to be significant when the *p* values were <0.05.

## 3. Results and discussion

### 3.1 HPLC chromatograms

**Fig. 1:**
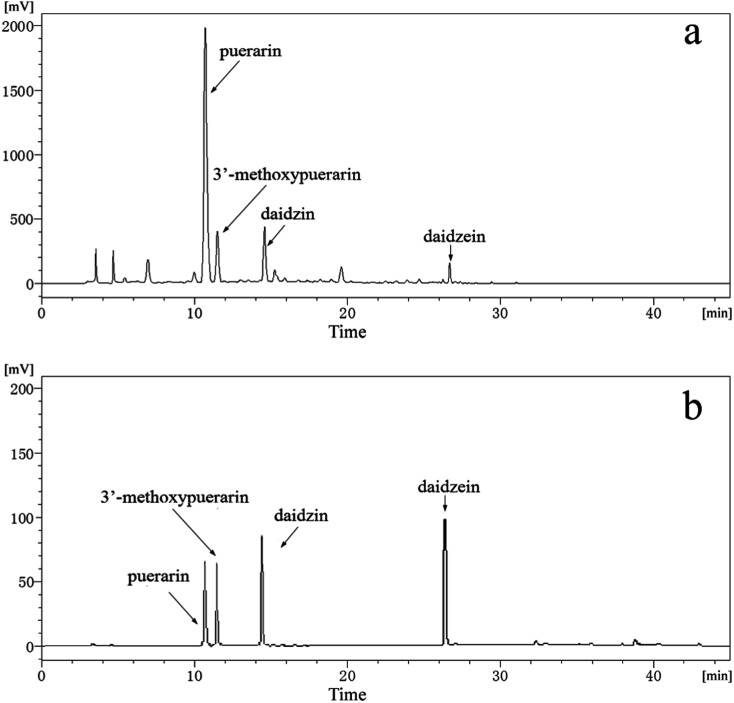
(a) Typical HPLC chromatogram of the *Puerariae lobata* extract obtained by SCWE (120 °C; 15 min; 4.50 mL water); (b) HPLC chromatogram of standard substances (puerarin, 3′-methoxypuerarin, daidzin and daidzein) mixed at concentrations of 10 μg mL^−1^.

Fig. 1b displays HPLC chromatograms of standard substances including puerarin, 3′-methoxypuerarin, daidzin and daidzein mixed at concentrations of 10 μg mL^−1^. As shown in Fig. 1b, the retention times of the four standard substances are 10.8, 11.8, 14.4 and 26.6 min, respectively. Fig. 1a shows a typical chromatogram of the *Puerariae lobata* extract obtained using SCWE at 120 °C for 15 min with 4.5 mL water. By comparing the retention times and UV spectra with those of the reference standards, four main peaks corresponding to puerarin, 3′-methoxypuerarin, daidzin and daidzein were identified. Other minor peaks were not identified in this study because of the lack of suitable standards. Thus, puerarin, 3′-methoxypuerarin, daidzin and daidzein were chosen as the objective compounds for the study.

### 3.2 Calculation of total isoflavone yield

The yields of puerarin, 3′-methoxypuerarin, daidzin and daidzein were obtained using the following five concentrations: 3.0, 6.0, 9.0, 12.0, and 15.0 μg mL^−1^. The standard curves obtained corresponded to *y* = 17 644*c*_1_ − 2725.7 (*R*^2^ = 0.9998) for puerarin; *y* = 21 514*c*_2_ − 4239.5 (*R*^2^ = 0.9996) for 3′-methoxypuerarin; *y* = 33 830*c*_3_ − 21 418 (*R*^2^ = 0.999) for daidzin; and *y* = 33 830*c*_4_ − 21 418 (*R*^2^ = 0.9991) for daidzein (where *y* is the peak area from HPLC chromatograms and *c* is the concentration of the target extracts). The extraction yields of the extracts were calculated with the following equation: 1
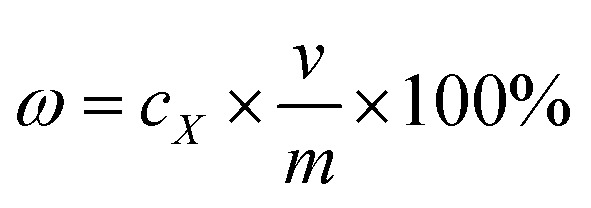
where *ω* is the extraction yield; *c*_*X*_ is the concentration of the target extract (*X* = 1, 2, 3, 4, corresponding to puerarin, 3′-methoxypuerarin, daidzin and daidzein, respectively); *v* is the final collection volume of the extract with methanol and *m* is the mass of *Puerariae lobata*. Herein, the total isoflavone yield was the summation of the extraction yields of puerarin, 3′-methoxypuerarin, daidzin and daidzein. The contents of all the extracts were measured using HPLC, and a spiking test was used to ensure that an accurate qualitative analysis was performed.

### 3.3 Effect of extraction temperature on the yields of the four isoflavones

**Fig. 2:**
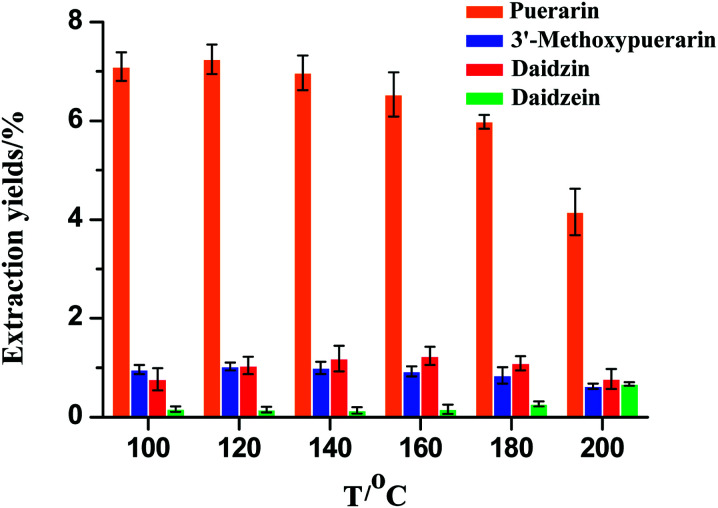
Effect of extraction temperature on the extraction yields of isoflavones from *Puerariae lobata* (30 min; 4.50 mL water).

The extraction temperature is an important factor that could influence the mass transfer rate and solubility of the isoflavones. As shown in Fig. 2, the extraction temperature range of 100–200 °C was chosen. When the extraction temperature increased from 100 to 120 °C, the extraction yields for puerarin and 3′-methoxypuerarin increased slightly. When the extraction temperature increased from 120 to 200 °C, the extraction yields for puerarin and 3′-methoxypuerarin decreased obviously. Daidzin showed a similar trend but reached a maximum at 160 °C. However, the extraction yield of daidzein remained constant from 100 to 160 °C and increased sharply when the extraction temperature exceeded 160 °C. From the structures of puerarin and 3′-methoxypuerarin, we could deduce that there was an 8-β-d-glucopyranoside bond between the isoflavone skeleton and glucoside, so it was facile to conclude that the glucopyranoside bond was unstable and began to break down when the extraction temperature exceeded 120 °C. As for daidzin, there was an oxygen bond between the isoflavone skeleton and glucoside. When the extraction temperature exceeded 160 °C, the oxygen bond became unstable and so the extraction yield of daidzin reached a maximum at 160 °C. In contrast there was a relatively stable isoflavone skeleton for daidzein. At 160 °C, daidzin degraded and produced daidzein, so the extraction yield of daidzein increased obviously.

### 3.4 Effect of extraction time on the yields of the four isoflavones

**Fig. 3:**
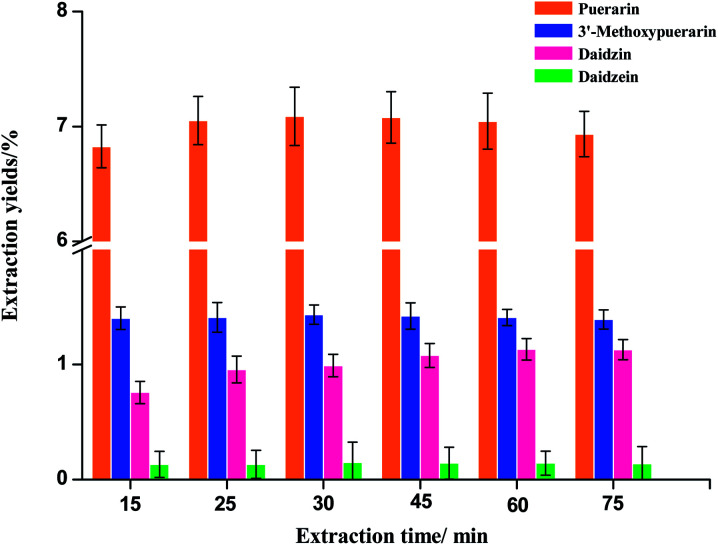
Effect of extraction time on the extraction yields of isoflavones from *Puerariae lobata* (120 °C; 4.50 mL water).

Extraction time is another important parameter for the extraction process. In the phase I experiment, *Puerariae lobata* powders were extracted at 120 °C for 15, 30, 45, 60 and 75 min, as shown in Fig. 3. In general, the extraction yields of puerarin, 3′-methoxypuerarin and daidzin significantly increased when the extraction time increased from 15 to 45 min. After 45 min, an extension of the extraction time could not significantly improve the extraction yields of puerarin, 3′-methoxypuerarin and daidzin, however, the extraction yield of daidzein actually increased a little. These results indicated that the optimal extraction time for the four main isoflavones was between 30 and 45 min.

### 3.5 Effect of solid/liquid ratio on the yields of the four isoflavones

**Fig. 4:**
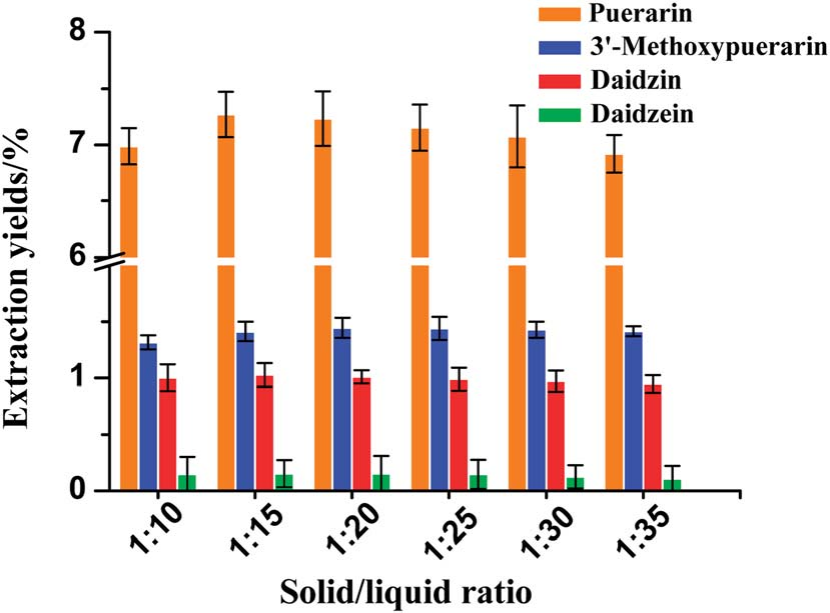
Effect of solid/liquid ratio on the extraction yields of isoflavones from *Puerariae lobata* (120 °C; 4.50 mL water).

The effect of various water loadings on the extraction yields of the four isoflavones is shown in Fig. 4. In general, the use of a solid/liquid ratio from 1 : 10 to 1 : 35 at 120 °C and 30 min had a small effect on the extraction yields of 3′-methoxypuerarin, daidzin and daidzein. The extraction yield of puerarin increased when the solid/liquid ratio increased from 1 : 10 to 1 : 15 and then decreased slightly when the solvent dosage increased over 1 : 15. The reason for the trend may be as follows: when the extract solvent volume increased to 4 mL, the isoflavones could be completely extracted. However, a further increase of the water volume could decrease the solubility of puerarin, 3′-methoxypuerarin and daidzein. Thus, the optimal solid/liquid ratio of 1 : 15 (g mL^−1^) was selected.

### 3.6 Parameters optimized by RSM for total isoflavones

**Fig. 5:**
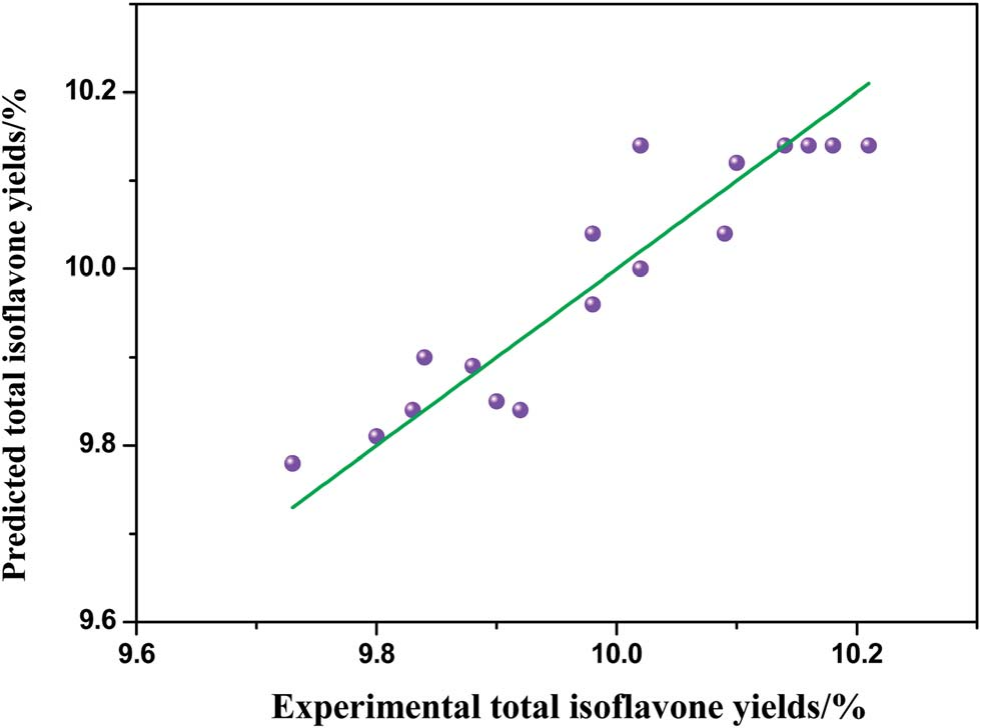
Comparison of experimental and predicted total isoflavones yields from *Puerariae lobata*.

To further study the interactions between the experimental factors, we optimized the extraction temperature, extraction time and solid/liquid ratio with RSM. The symbols and levels are shown in Table 1. The results are given in Table 2. The experimental values of extraction yield of total isoflavones from *Puerariae lobata* were analyzed by multiple regressions to fit the second order regression equation, and the regression model in terms of coded factors was predicted as follows (Table 4, eqn (2)):

2 *Y* = 10.16 + 0.0075*x*_1_ + 0.096*x*_2_ + 0.026*x*_3_ − 0.021*x*_1_*x*_2_ + 0.003632*x*_1_*x*_3_ − 0.066*x*_2_*x*_3_ − 0.18*x*_1_^2^ − 0.054*x*_2_^2^ − 0.11*x*_3_^2^

where *Y* is the response value (the extraction yields of total isoflavones from *Puerariae lobata*) and *x*_1_, *x*_2_ and *x*_3_ are shown in Table 2. The significance of each coefficient was determined by using the *F*-test and *p*-value illustrated in Table 3. For any of the terms in the models, a large *F*-value and a small *p*-value indicated a more significant effect on the respective response variables. The parity plot in Fig. 5 shows a comparison between the experimental yields of total isoflavones from *Puerariae lobata* and the values calculated by eqn (2). A perfect fit of the model to the data would result in all points lying on the diagonal in Fig. 5. The coefficient of determination of 0.927 indicates that the model demonstrates a accurate result, with reasonable scatter around the diagonal and no trends in the residuals.

**Table 1:** Experimental domain with natural and coded values of independent variables used in Box–Behnken design (BBD)

Factors	Units	Variables	Range and levels		
			−1	0	1
Extraction temperature	°C	*x*_1_	110	120	140
Extraction time	min	*x*_2_	15	30	45
Liquid/solid ratio	g mL^−1^	*x*_3_	1 : 10	1 : 15	1 : 25

**Table 2:** Box–Behnken program experimental design with natural and coded SCWE conditions and experimentally obtained values for total yields of isoflavones from *Puerariae lobata*

Run	Temperature	Time	Liquid/solid ratio	Yield (%)	
	*x*_1_ (°C)	*x*_2_ (min)	*x*_3_ (g mL^−1^)	Actual value	Predicted value
1	1	0	1	9.83 ±0.31	9.84 ± 0.30
2	0	0	0	10.02 ± 0.22	10.14 ± 0.21
3	0	0	0	10.14 ± 0.32	10.14 ± 0.33
4	1	0	−1	9.84 ± 0.47	9.90 ± 0.48
5	−1	−1	0	9.80 ± 0.45	9.81 ± 0.49
6	0	0	0	10.16 ± 0.39	10.14 ± 0.40
7	0	1	1	10.10 ± 0.41	10.12 ± 0.40
8	0	0	0	10.18 ± 0.37	10.14 ± 0.36
9	1	1	0	10.02 ± 0.44	10.00 ± 0.43
10	0	−1	−1	9.98 ± 0.24	9.96 ± 0.23
11	0	0	0	10.21 ± 0.30	10.14 ± 0.29
12	0	1	−1	10.09 ± 0.47	10.04 ± 0.46
13	−1	1	0	9.98 ± 0.29	10.04 ± 0.29
14	0	−1	1	9.73 ± 0.18	9.78 ± 0.17
15	1	−1	0	9.90 ± 0.31	9.85 ± 0.30
16	−1	0	1	9.92 ± 0.43	9.84 ± 0.42
17	−1	0	−1	9.88 ± 0.27	9.89 ± 0.26

**Table 3:** Analysis of variance of the fitted second-order polynomial model for total extraction yields of isoflavones

Source	Sum of squares	Mean df	*F* square	*p*-Value	Prob > *F*
Model	0.3	9	0.033	5.35	0.0189
*x*_1_	4.50 × 10^−6^	1	4.5 × 10^−6^	7.28 × 10^−4^	0.9792
*x*_2_	0.07	1	0.07	11.34	0.012
*x*_3_	5.18 × 10^−3^	1	5.18 × 10^−3^	0.84	0.3905
*x*_1_ *x*_2_	1.81 × 10^−3^	1	1.81 × 10^−3^	0.29	0.6056
*x*_1_ *x*_3_	5.57 × 10^−5^	1	5.57 × 10^−5^	9.01 × 10^−3^	0.9271
*x*_2_ *x*_3_	0.017	1	0.017	2.82	0.1371
*x*_1_^2^	0.1	1	0.1	16.89	0.0045
*x*_2_^2^	0.012	1	0.012	2.01	0.1994
*x*_3_^2^	0.055	1	0.055	8.82	0.0208
Residual	0.043	7	6.18 × 10^−3^	—	—
Lack of fit	0.022	3	7.28 × 10^−3^	1.36	0.3752
Pure error	0.021	4	5.36 × 10^−3^	—	—
Cor total	0.34	16	—	—	—

**Table 4:** The predicted regression model in terms of coded factors from Box–Behnken program analysis

Factor	Coefficient estimate	df	Standard error	95% CI low	95% CI high	VIF
Intercept	10.16	1	0.043	10.06	10.27	
*x*_1_	0.0075	1	0.031	−0.06	0.087	1.06
*x*_2_	0.096	1	0.032	6.98 × 10^−3^	0.16	1.05
*x*_3_	0.026	1	0.032	−0.053	0.097	1.05
*x*_1_ *x*_2_	−0.021	1	0.043	−0.14	0.057	1.05
*x*_1_ *x*_3_	0.003632	1	0.043	−0.094	0.11	1.05
*x*_2_ *x*_3_	−0.066	1	0.044	−0.17	0.038	1
*x*_1_^2^	−0.18	1	0.049	−0.3	−0.064	1.06
*x*_2_^2^	−0.054	1	0.043	-0.14	0.062	1.01
*x*_3_^2^	−0.11	1	0.043	-0.23	−0.028	1.01

**Fig. 6:**
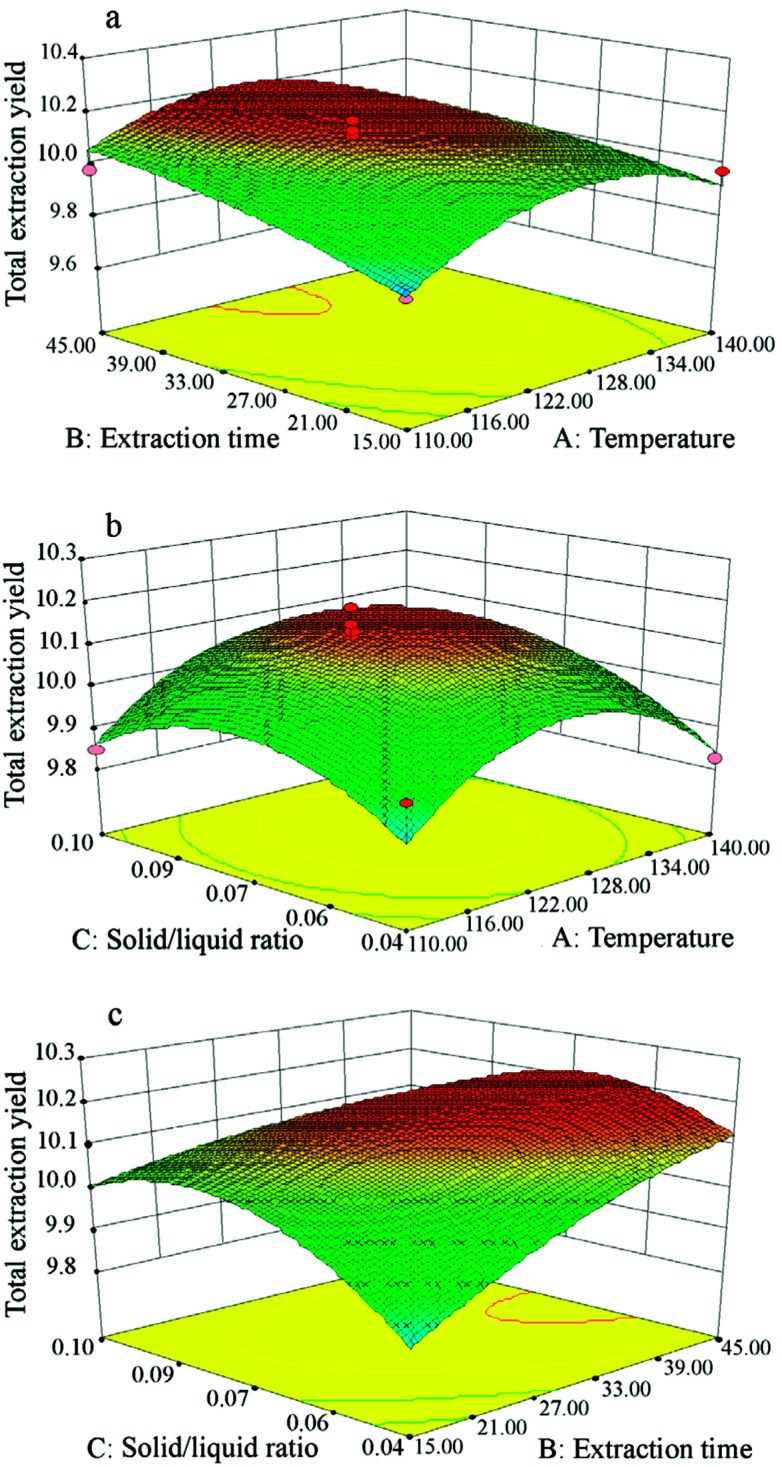
Response surface plots showing interactions between experimental parameters on the total isoflavone yields from *Puerariae lobata*: (a) extraction time (min) and temperature (°C); (b) solid/liquid ratio and temperature (°C); (c) solid/liquid ratio and extraction time (min).

To consider the interactions between independent variables, 3D response surface plots are depicted in Fig. 6, showing how pairs of extraction parameters affect the extraction yields of total isoflavones from *Puerariae lobata*. All of the three surfaces are saddle-shaped, with a maximum point in the center of the experimental domain. Based on the 3D response surface plots, the optimum conditions for *Puerariae lobata* extraction were calculated to be as follows: extraction temperature of 120 °C, extraction time of 45 min and solid/liquid ratio of 1 : 15. Under these optimum extraction conditions, the total isoflavone yields of three replicates were 10.21 ± 0.34%, 9.91 ± 0.41% and 10.03 ± 0.51%, and the average was 10.05 ± 0.42%, which is very close to the predicted value of 10.04 ± 0.41%; this demonstrates that the model showed a good fit with the experimental values.

### 3.7 Comparison of different extraction methods on the total isoflavone yields from *Puerariae lobata*

To investigate the extraction effect of subcritical water, the extraction results obtained using this process were compared with those obtained by 70% ethanol reflux and ultrasonic extraction. Table 5 shows the extraction conditions and total isoflavone extraction yields. It can be seen that the extraction yields with SCWE were the highest, but reflux and ultrasonic extraction had extraction yields that were almost as high. The extraction time for SCWE was shorter than those for reflux and ultrasonic extraction. Besides, the consumption of extraction solvent by SCWE was less than those for the conventional methods. This is probably due to the more complete diffusion of water molecules into the particles of the herbal materials. Therefore, it is clear that subcritical water is an effective extraction solvent and that SCWE could utilize less solvent and take a shorter time than other extraction methods. In addition, as shown in Table 5, no significant differences in the isoflavone yields produced by the three different methods were found.

**Table 5:** Comparison of the total isoflavone yields obtained from *Puerariae lobata* with different extraction methods

Extraction method	Operation conditions			Extraction yields
Reflux extraction	90 °C	120 min	1 : 40	9.21 ± 0.36%
Ultrasonic extraction	50 °C	45 min	1 : 30	9.64 ± 0.23%
SCWE	120 °C	35 min	1 : 15	10.05 ± 0.42%

## 4. Conclusion

In this study, the extraction from *Puerariae lobata* of four individual isoflavones, namely puerarin, 3′-methoxypuerarin, daidzin and daidzein, as well as that of the total isoflavones were studied using subcritical water. The extraction temperature, extraction time and solid/liquid ratio were optimized by single-factor experiments for the four isoflavones and the total isoflavone yield was optimized by RSM. Compared with conventional extraction methods, SCWE provides a higher extraction efficiency due to its high diffusion velocity. The presented results demonstrate that subcritical water is an excellent extractant and that SCWE is a simple, fast, environmentally friendly extraction method which has a low consumption of solvent.

## Conflicts of interest

The authors declare that there is no conflict of interest regarding the publication of this paper.

## Supplementary Material

## References

[cit1] Chen Y. G., Song Y. L., Wang Y., Yuan Y. F., Huang X. J., Ye W. C., Wang Y. T., Zhang Q. W. (2014). J. Pharm. Biomed. Anal..

[cit2] Cherdshewasart W., Sutjit W. (2008). Phytomedicine.

[cit3] Xu M. E., Xiao S. Z., Sun Y. H., Zheng X. X., Ou-Yang Y., Guanm C. (2005). Life Sci..

[cit4] Boue S. M., Wiese T. E., Nehls S., Burow M. E., Elliott S., Carter-Wientjes C. H., Shih B. Y., Mclachlan J. A., Cleveland T. E. (2003). J. Agric. Food Chem..

[cit5] Hsu F. L., Liu I. M., Kuo D. H., Chen W. C., Su H. C., Cheng J. T. (2003). J. Nat. Prod..

[cit6] Jiang R. W., Lau K. M., Lam H. M., Yam W. S., Leung L. K., Choi K. L., Waye M. M. Y., Mak T. C. W., Woo K. S., Fung K. P. (2005). J. Ethnopharmacol..

[cit7] Ru S. G., Chen D., Wang W. (2015). Period. Ocean Univ. China.

[cit8] Eun J. S., Kyung S. Y., Byung-Sun M. (2012). Arch. Pharmacal Res..

[cit9] Jin S. E., Son Y. K., Min B., Jung H. A., Choi J. S. (2012). Arch. Pharmacal Res..

[cit10] Wang Y., Yin L., Li Y., Liu P., Cui Q. (2008). Clin. Orthop. Relat. Res..

[cit11] Mei-Hwa L., Chuan-Chuan L. (2007). Food Chem..

[cit12] Yasuda T., Endo M., Kon-no T., Kato T., Mitsuzuka M., Ohsawa K. (2005). Biol. Pharm. Bull..

[cit13] Dong W. L., Changho L., In-Ho K., Yun T. K. (2013). Molecules.

[cit14] Min-Jung K., Chan-Ick C., Sang-Woo C., Myong-Soo C. (2011). J. Food Eng..

[cit15] He J. T., Zhao Z. W., Shi Z. H., Zhao M. P., Li Y. Z., Chang W. B. (2005). J. Agric. Food Chem..

[cit16] Gbashi S., Adebo O. A., Piater L., Madala N. E., Njobeh P. B. (2017). Sep. Purif. Rev..

[cit17] Bianca D., Jeremy J., Jamuna C., Liam K. B., James N. S., Nathan K., Miguel S., Nuri G., Alex B., Jason S. (2017). ChemistrySelect.

[cit18] Kim W. J., Veriansyah B., Lee Y. W., Kim J., Kim J. D. (2010). J. Ind. Eng. Chem..

[cit19] Allmon S. D., Dorsey J. G. (2010). J. Chromatogr. A.

[cit20] Liu F., Ong E. S., Li S. F. Y. (2013). Food Chem..

[cit21] Ibaňez E., Kubátová A., Seňoráns F. J., Cavero S., Reglero G., Hawthorne S. B. (2003). J. Agric. Food Chem..

[cit22] Zekovic Z., Vidovic S., Vladic J., Radosavljevic R., Cvejin A., Mohamed P. (2014). J. Supercrit. Fluids.

[cit23] Derringer G. (1980). Journal of Quality Technology.

[cit24] Bas D. (2007). J. Food Eng..

[cit25] Gan C. Y., Latiff A. A. (2011). Food Chem..

[cit26] Bezerra M. A., Santelli R. E., Oliveira E. P., Villar L. S., Escaleira L. A. (2008). Talanta.

[cit27] Hisashi M., Toshio M., Fengming X., Kiyofumi N., Masayuki Y., Yan B., Xing D. M., Ding Y., Tao J. L., Du L. J. (2005). J. Pharm. Biomed. Anal..

[cit28] Zhu D. Y. (2015). China Brewing.

[cit29] Li H. T. , Study on extraction technology of effective components of *Puerariae* and its antialcoholic effect, Northeast Agricultural University, Harbin, 2006

